# Fabrication of Stainless Steel/Alumina Composite Powders by Spray Granulation and Plasma Spheroidization

**DOI:** 10.3390/ma18081872

**Published:** 2025-04-19

**Authors:** Elodie Cabrol, Sandrine Cottrino, Hocine Si-Mohand, Gilbert Fantozzi

**Affiliations:** 1 University of Lyon, Ecole Centrale de Lyon, CNRS, ENTPE, Laboratoire de Tribologie et Dynamique des Systèmes, UMR5513, ENISE, 42023 Saint Etienne, France; hocine.si-mohand@enise.fr; 2 MATEIS, INSA-Université Lyon1-CNRS, UMR 5510, INSA de Lyon, 69621 Villeurbanne, France; sandrine.cottrino@insa-lyon.fr (S.C.); gilbert.fantozzi@insa-lyon.fr (G.F.)

**Keywords:** spray granulation, plasma spheroidization, composite powder, cermet powder, spark plasma sintering, 316L stainless steel/alumina

## Abstract

This work presents a new approach for the fabrication of 316L/Al_2_O_3_ composites, based on a combination of spray granulation, radio frequency (RF) plasma spheroidization and spark plasma sintering (SPS). Initially, a suspension containing 316L and alumina powders is formulated by precisely adjusting the pH and selecting an appropriate dispersant, thereby ensuring homogeneous dispersion of the constituents. The spray granulation process then produces granules with controlled size and morphology. RF plasma spheroidization, carried out using a TekSphero-40 system, is investigated by varying parameters such as the power, gas flow rates, injection position and feed rate, in order to optimize the formation of spherical and dense particles. The analysis reveals a marked sensitivity to heat transfer from the plasma to the particles, with a tendency for fine particles to segregate, which underscores the necessity for precise control of the processing conditions. Finally, SPS densification, performed under a constant pressure and a rigorously controlled thermal cycle, yields composites with excellent density and hardness characteristics. This study thus demonstrates that the proposed hybrid process offers an optimal synergy between a uniform distribution of alumina and a controlled microstructure, opening up promising avenues for the design of high-performance composite materials for demanding applications.

## 1. Introduction

Austenitic stainless steels exhibit a good resistance to corrosion and high ductility, and are used for many applications, such as in the aerospace, automotive, marine, defence [1] and chemical industries, and in medical applications [2,3]. However, wider use of these steels requires improved mechanical properties and wear resistance [4]. These improvements can be achieved by using metal matrix composites reinforced with ceramic particles, which provide higher hardness, toughness, wear resistance and corrosion resistance. Several types of ceramic particles have been used [5,6], including Al_2_O_3_, SiC, TiC, TiN and ZrC.

It has been reported that, for 316L/Al_2_O_3_ composites fabricated by hot pressing, increasing the reinforcement content of alumina within the 316L matrix significantly enhances the material hardness, from 153 HB at 0 wt% Al_2_O_3_, to 270 HB at 15 wt% [6]. In the case of functionally graded materials (FGMs) based on 316L/Al_2_O_3_, consisting of layers containing 5, 10 and 15 wt% Al_2_O_3_, the microhardness increases progressively with the reinforcement content, reaching a maximum of 367 HV in the layer containing 15 wt% Al_2_O_3_ [3].

In terms of wear performance, the addition of 50 wt% Al_2_O_3_ particles has been shown to lead to an approximate 86% reduction in wear rate, with a 7.2-fold decrease in volumetric loss compared to 316L [6]. Moreover, an increased alumina content has also been associated with improved corrosion resistance of 316L/Al_2_O_3_ composites [6]. A comparable trend was observed in composites reinforced with yttria-stabilized zirconia (YSZ), where optimal corrosion resistance was achieved at 5 wt% YSZ content [7].

In addition, the incorporation of 2 wt% niobium carbide (NbC) has been shown to significantly enhance both the mechanical properties and the wear resistance of 316L stainless steel produced by laser powder bed fusion (L-PBF) [8]. Similarly, the introduction of titanium carbide (TiC) into the 316L matrix has been shown to improve the mechanical, tribological and corrosion performance of 316L/TiC composites produced by L-PBF. At an optimal content of 2 wt% TiC, the average microhardness increased by 12.4% (from 298 HV_0.2_ to 335 HV_0.2_), while the average coefficient of friction decreased to 0.12, in comparison with 0.37 for unreinforced 316L stainless steel [9].

Alumina is attractive for metal matrix composites, due to its good hardness, wear resistance, chemical inertness, thermal stability and low cost.

Several techniques can be used to produce these composites:

- Melting and solidification (conventional ingot casting [10], laser powder bed fusion [11]).
- A powder metallurgy process with the following steps: powder mixing, powder preparation, debinding and sintering. In order to form the ceramic powder into the component shapes, uniaxial compaction is generally used; metal injection moulding can also be employed [12]. Different sintering processes are possible: pressureless sintering [3,5], hot pressing [6] and spark plasma sintering (SPS) [13,14].

In these processes, the powders employed typically consist of blends of both materials. Such mixtures exhibit heterogeneities arising from particle agglomeration, particularly when the alumina powder used is nanoscale. Post-consolidation, the addition of Al_2_O_3_ to 316L results in increased hardness, but also a deterioration in mechanical strength and elongation at break, due to porosity formation and the poor wettability of 316L with Al_2_O_3_ [15,16].

To address these challenges of powder mixture heterogeneity, several studies have adopted a two-step approach: granule preparation via spray granulation, followed by RF plasma spheroidization. This methodology has been successfully applied to fabricate composite powders, at the particle scale, of metal/metal [17,18,19], metal/ceramic [20,21,22], ceramic/ceramic [23,24] or more complex compositions, such as Dual-Phase High-Entropy Ceramics (DPHECs) [25].

Only a limited number of metal–ceramic composites have been synthesized using the combined process of spray granulation and RF plasma spheroidization. In the case of WC–Co composites, granules are produced by mixing nanopowders of W, C and Co in an aqueous suspension [21]. These granules are subsequently spheroidized using a 30 kW RF induction plasma torch operated under an argon atmosphere containing 3 vol% hydrogen. The resulting particles, with an average size ranging from 17 to 20 µm, exhibit chemical modifications characterized by a reduction in both carbon and cobalt content. For the W–ZrC composite powder [22], granules are formulated from a 3 µm tungsten powder and 50 nm zirconium carbide (ZrC) nanopowder. After plasma spheroidization, the resulting W–ZrC powder exhibits a spherical morphology with an average particle size of approximately 25 µm. ZrC is uniformly distributed within the spheroidized particles. However, a decrease in ZrC content is observed, ranging from 0.5 to 1 wt.% relative to the initial formulation.

In this study, 316L/Al_2_O_3_ composite granules were prepared by spray granulation and spheroidized using RF plasma. The resultant powders were subsequently consolidated using spark plasma sintering (SPS); their density, hardness and microstructure were determined. This enabled a comparative evaluation of materials obtained from plasma-processed powders against those fabricated from granules and powder blends.

## 2. Materials and Methods

### 2.1. Initial Powders

The composite used in this paper was processed using a mixture of two commercial powders: high-purity (99.9%) commercial alumina Al_2_O_3_ (P172HPB—ALTEO, Gardanne, France) with a median nanoparticle size of 400 nm, obtained by the Bayer process, and 316L powder (PF-3K—EPSON ATMIX, Hachinohe, Japanalteo) with a median microparticle size of 3 µm, obtained by the spinning water atomization process.

### 2.2. Suspension and Spray Granulation

A formulation was prepared using a mixture of the two powders (80 vol% 316L/20 vol% Al_2_O_3_), with polyacrylic acid as a dispersant (PAA, Mw 2000). The dispersant was dissolved (0.5 wt%) in high-purity water after adjusting the pH to 11. The solution was homogenized for 10 min before adding the alumina powder. The alumina suspension was stirred for 24 h before the steel powder was added. The mixture was then stirred for a further 4 h before being atomized.

Wet laser granulometry (Malvern—Scirocco 2000, Worcestershire, UK) was used to check the state of dispersion.

The resulting slurry, with a solid loading ratio of 50 wt%, was spray-dried in an ultrasonic atomizer (Synetude SAS, Cognin, France). A 20 kHz ultrasonic probe was used, with inlet and outlet temperatures maintained at 250 °C and 110 °C, respectively.

The granules were then treated for 1 h at 400 °C for debinding, and for 1 h at 800 °C for consolidation.

### 2.3. Plasma Spheroidization

**Figure 1:**
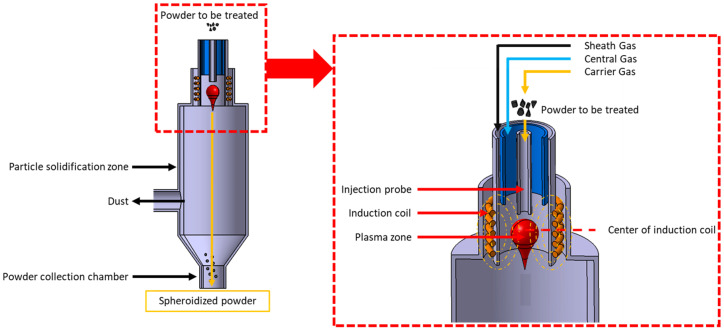
Schematic diagram of plasma spheroidization [26].

The granules were processed using RF plasma treatment on a TekSphero-40 spheroidization system (TEKNA, Sherbrooke, QC, Canada). This treatment was employed to produce spherical and dense 316L/Al_2_O_3_ composite particles. A schematic representation of the spheroidization process, previously described in the literature [26], is presented in Figure 1.

The TekSphero-40 process parameters include the power, gas flow rates, powder feed rate and position of the powder injection probe in the plasma. The power can range from 20 to 40 kW. Argon gas is used to generate the plasma (central gas), convey the powder (carrier gas) and protect the torch walls (sheath gas). Hydrogen is often mixed with the sheath gas to enhance the thermal conductivity of the plasma. The atmosphere within the torch was maintained at a slight overpressure (15 psi) to prevent air entering. The processed powder was collected under argon, and subsequently washed with water to remove the nanoparticles on the surface of the micrometric particles. The parameters employed in this study are detailed in Table 1 and Table 2.

### 2.4. Characterization of Spray-Granulated and Spheroidized Granules

The granule bulk density was obtained by helium pycnometry (Micromeritics—Accpyc 1340, Mérignac, France). The size distribution of the granules was measured by laser granulometry (Malvern—Scirocco 2000, Worcestershire, UK). All granule batches were investigated by scanning electron microscopy (SEM) using a Zeiss-Supra 55 (Rueil Malmaison, France).

In order to estimate the average size of the inner granule voids, X-ray computed microtomography scans were conducted on bulk samples. This technique is an alternative way to determine the pore size and visualize the microstructure of an entire granule, with a resolution comparable to that of optical microscopy instruments, and operating in a non-destructive manner. As a result, individual pores were visualized and the true internal morphology of granules was revealed. X-ray tomography was performed using a v/tome/x (Phoenix X-ray Company, Villebon-sur-Yvette, France) X-ray microtomograph. This commercial device includes a nanofocus transmission X-ray tube (W target).

### 2.5. Densification

The composites were densified using the spark plasma sintering (SPS) technique. The granules were introduced into a graphite die that allowed uniaxial stress to be applied during sintering. A pulsed direct electrical current was applied via electrodes and passed through the pressing chamber to heat the sample. The temperature was measured using an optical pyrometer positioned close to the sample. A graphite paper foil was placed between the die, pistons and powder to protect the pressing elements.

The graphite die had an internal diameter of 10 mm and a thickness of 10 mm. During the entire cycle, a constant pressure of 64 MPa (5 kN) was maintained, and sintering was performed under a high vacuum (∼1 Pa). The temperature cycle used was as follows: heating up to 1040 °C at a ramping rate of 100 °C/min; heating up to 1070 °C at a rate of 10 °C/min, to avoid overshoot; maintenance at 1070 °C for 1 h; and cooling at a rate of 50 °C/min.

### 2.6. Characterization of Sintered Samples

The density of sintered samples was measured using the Archimedes method, with distilled water. The values of the relative densities were calculated, assuming a theoretical density of 7.17 g/cm^3^, and taking into account the ratio of both materials and a theoretical density of 7.98 g/cm^3^ for the 316L and 3.94 g/cm^3^ for alumina. The measurement uncertainty was ± 0.2.

All sintered bodies were observed by scanning electron microscopy (SEM) using a Zeiss-Supra 55VP, after a polishing step. The polishing process proceeded as follows: first, diamond shields with grit sizes of 200, 500 and 1200 µm were used. Then, diamond grains suspended in a lubricating fluid were applied with polishing cloths with grit sizes of 6 nm, 3 nm and 1 nm. The polishing effect was continuously controlled after each step to obtain the highest-quality mirror-finished samples.

A conventional Vickers hardness test (TestWell FV-700 equipment, Gennevilliers, France) was performed with a 5 kg load applied for 10 s. The choice of the applied load was made to ensure a well-defined imprint shape and a surface representative of the composite structure.

## 3. Results and Discussion

### 3.1. Initial Powders

**Figure 2:**
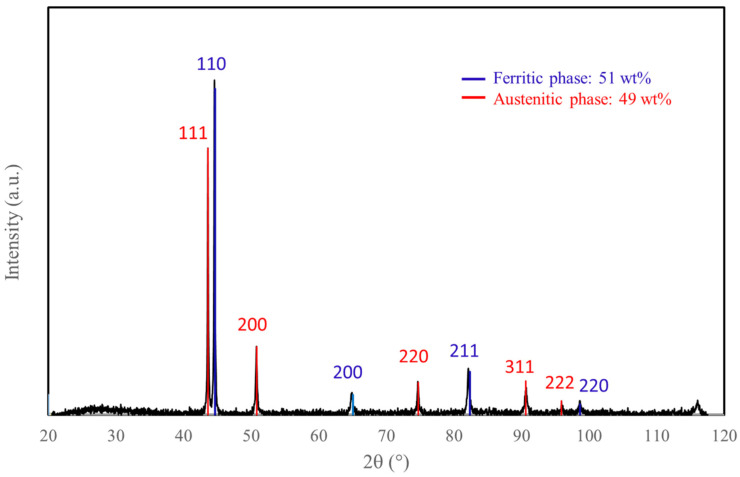
XRD analysis of 316L powder.

**Figure 3:**
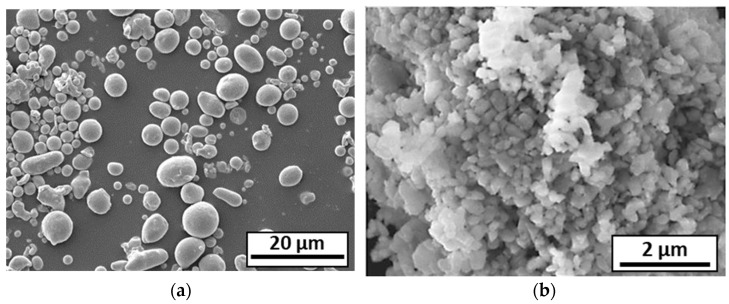
SEM observation of (**a**) 316L powder and (**b**) alumina powder.

**Figure 4:**
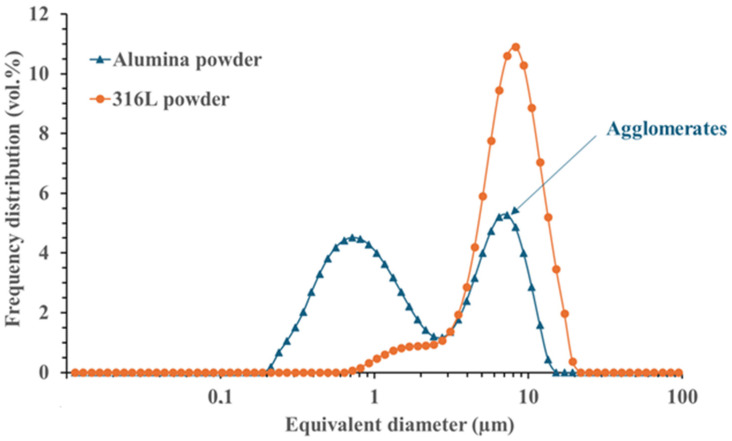
Particle size distribution of initial powders.

XRD analysis of the 316L powder showed that it was composed of two phases: ferrite and austenite (Figure 2). Figure 3a shows that the 316L powder is composed of spherical micronic particles, whereas the submicron alumina particles display more non-spherical and agglomerated nanoparticles (Figure 3b). A size distribution measurement was carried out on each of the initial powders (Figure 4): 316L powder with D10 = 1.4 µm/D50 = 3.6 µm/D90 = 8.1 µm, and alumina powder with D10 = 0.4 µm/D50 = 1.6 µm/D90 = 8.4 µm.

### 3.2. Spray Granulation Optimization

Several slurries were developed to optimize suspension parameters such as the solid loading ratio, dispersant ratio and nature (polyethylene glycol (PEG), polyvinyl alcohol (PVA), polyacrylic acid (PAA)) or pH (Table 3).

To determine the optimum parameters, the slurries and the granules obtained from these different slurries were characterized.

A first check was always conducted on the state of dispersion of the alumina alone, using the values given in the data sheet. In the case of slurry 3, it can be seen that the alumina disperses well, and that after the addition of the 316L powder, the slurry remains fluid and relatively stable.

**Figure 5:**
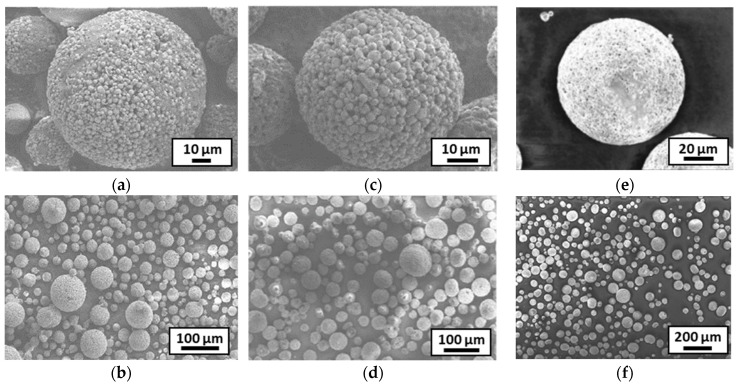
SEM observations of the different granule batches: (**a**,**b**) for slurry 1, (**c**,**d**) for slurry 2, (**e**,**f**) for slurry 3

**Figure 6:**
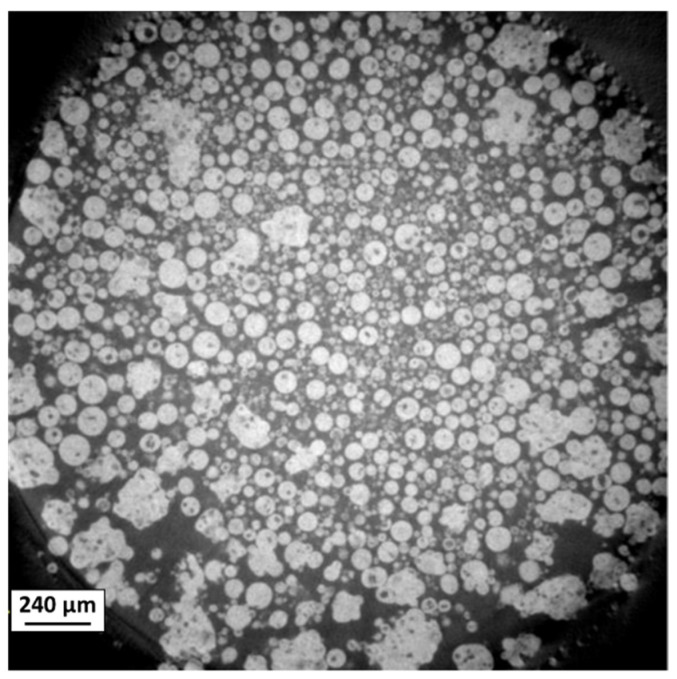
X-ray tomography slide of batch 1 granules.

The microstructure of the granules obtained from each slurry was observed by SEM and X-ray tomography (Figure 5 and Figure 6). The different batches of granules had a fairly wide size distribution, which was characterized by laser granulometry (Table 4). The granules appeared to be fairly homogeneous, consisting of a mixture of submicron alumina particles and micron stainless steel particles. Examination of the internal microstructure of batch 1 granules using X-ray tomography (Figure 6) revealed that some were hollow, but overall, they were mostly dense.

Slurry 3 seemed to give denser granules, closer to the theoretical density of the composite (theoretical density for a composite of 80 vol% 316L/20 vol% Al_2_O_3_: 7.17 g/cm^3^). This could be explained by its higher solid content. In addition, the size distribution was narrower, with the largest granules decreasing in size. The reduction in size of the larger granules was confirmed by SEM images (Figure 5).

### 3.3. Plasma Spheroidization

**Figure 7:**
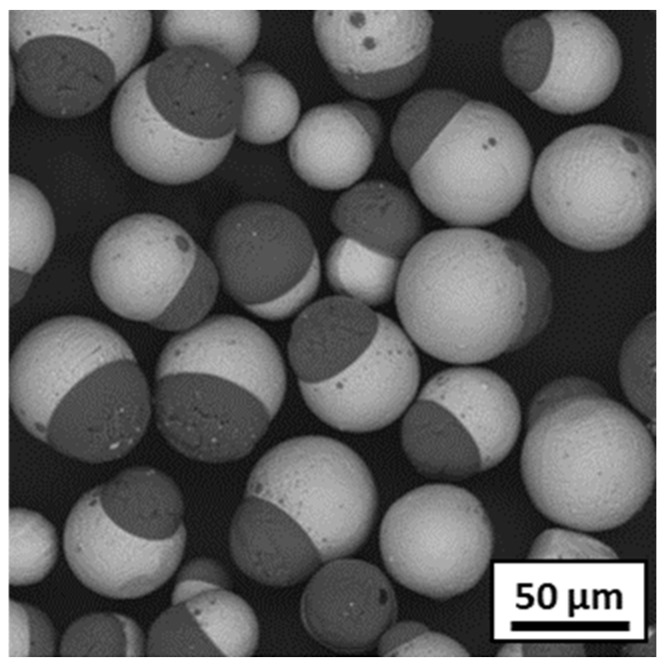
SEM observation of segregation between alumina (in black) and steel (in grey) after preliminary spheroidization tests (image given for test 3).

A parametric study was conducted by varying the power, gas composition, powder feed rate and injection position of the powder. The results demonstrated that, for all parameter sets applied in the preliminary tests (Table 1), a clear separation of alumina from steel was observed. The resulting particles were spherical and dense, with one side consisting of Al_2_O_3_ and the other of 316L (Figure 7).

The primary challenge in producing homogeneous 316L/Al_2_O_3_ composite particles arises from the poor wettability of metals and oxide ceramics at high temperatures [27]. To address this issue, we attempted to reduce the energy transferred to the particles, which is influenced by the plasma temperature (power, gas type and flow rates, powder feed rate) and the particle exposure time in the plasma (gas type and flow rates, injection probe position, powder feed rate) [28]. The goal was to prevent constituent segregation by initiating rapid cooling immediately after melting.

**Figure 8:**
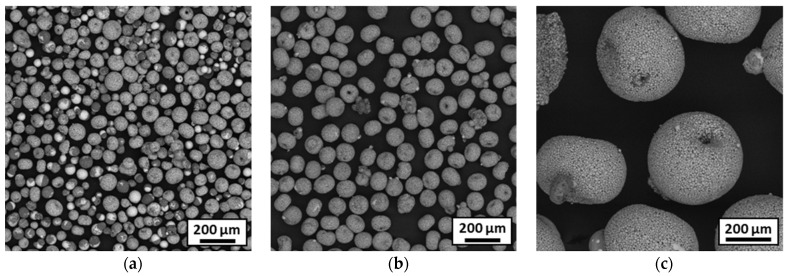
SEM observations of particles after optimal plasma treatment for different particle size (Ø): (**a**) for Ø < 106 µm, (**b**) for 90 µm < Ø < 106 µm and (**c**) for 90 µm < Ø < 106 µm

The optimal parameters for plasma treatment of 316L/Al_2_O_3_ granules are detailed in Table 2. The treatment exhibited varying effects depending on particle size. As shown in Figure 8a, fine particles consistently displayed segregation, while larger particles were homogeneous. This discrepancy is attributed to the wide particle size distribution in the granules: the energy required for melting larger particles is excessive for smaller ones, thereby triggering segregation in the latter. As a result, it was not feasible to treat the entire powder batch uniformly. Post-spheroidization, the powder was sieved at 90 µm to isolate particles with homogeneous alumina and steel distributions (Figure 8b,c).

### 3.4. Microstructure and Hardness of Sintered Composites

#### 3.4.1. Sintered 316L Stainless Steel

**Figure 9:**
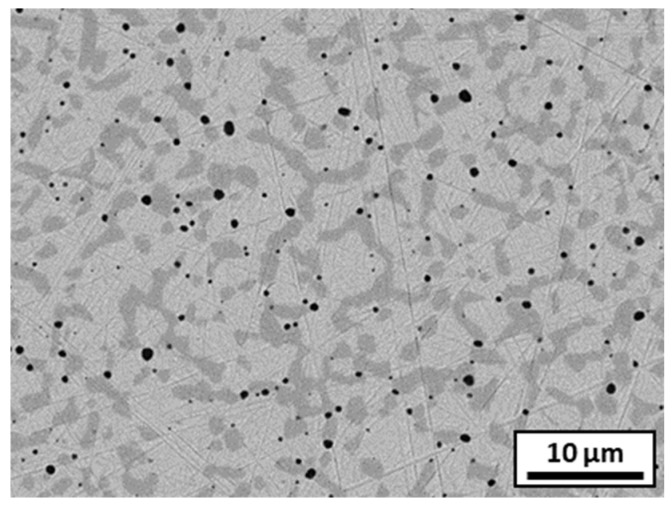
SEM microstructure of sintered 316L stainless steel.

To optimize the densification of the composite, the sintering parameters were determined from a sintered a stainless steel sample. This sample was characterized by XRD analysis, as well as by density and hardness measurements. The presence of two phases was observed: in dark grey, the ferritic phase, and in light grey, the austenitic phase (Figure 9). Pores with sizes in the µm range were also noted. The density was 99.5% of the theoretical density, and the hardness was 3.7 GPa.

#### 3.4.2. Composite Sintered from Ball-Milled Mixture

**Figure 10:**
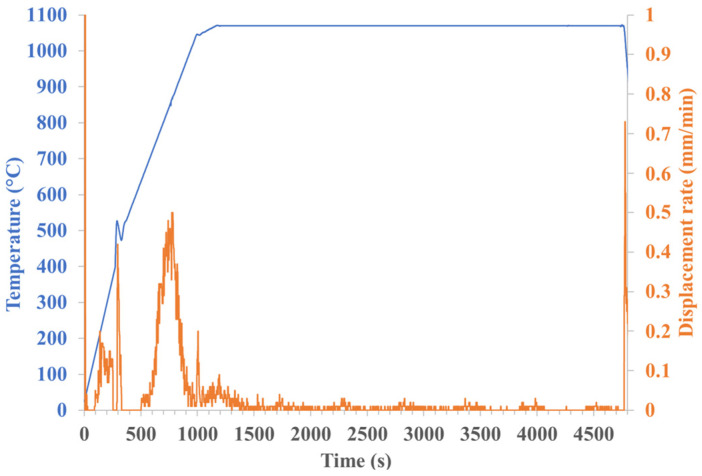
Evolution of temperature and piston displacement during sintering treatment of composite (80 vol% 316L/20 vol% Al_2_O_3_).

An initial composite was obtained by mixing the two powders using a dry ball-milling method, and then sintered. Figure 10 shows the evolution of piston displacement speed and temperature as a function of time for an 80 vol% 316L/20 vol% Al_2_O_3_ composite. Initially, there is a densification zone corresponding to particle rearrangement (between 0 and 300 s), followed by a sintering zone, with a significant densification peak at around 800 °C.

**Figure 11:**
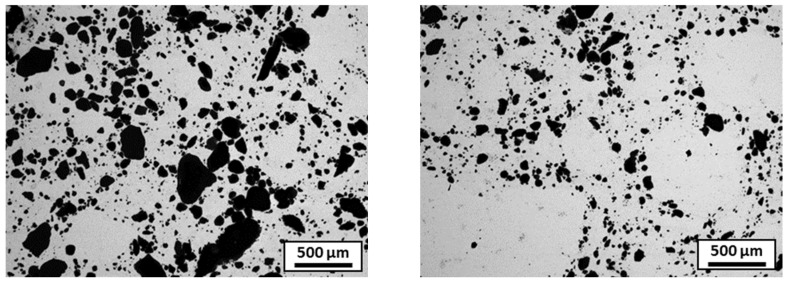
SEM microstructure of composite sintered from ball-milled mixture (alumina in black and steel in grey).

Figure 11 shows the microstructure of a ball-milled composite. It appears that the alumina powder is not well distributed; instead, it is agglomerated in clusters. The density is only 91.8% of the theoretical density of the composite, and the hardness 4.3 GPa.

#### 3.4.3. Composite Sintered from Spray-Granulated Granules

**Figure 12:**
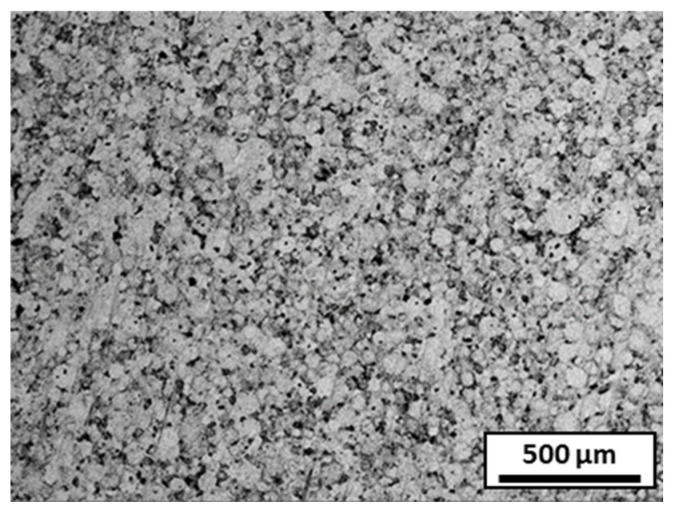
SEM microstructure of composite sintered from spray-granulated granules; alumina is in black and stainless steel is in grey.

The granules from slurry 3 were sintered using the same sintering parameters. The microstructures of the sintered samples are shown in Figure 12. The distribution of alumina within a granule was more or less homogeneous. However, after sintering, traces of the granules obtained by atomization were still visible; the granules were not properly destroyed. This explains the lower densification of the sintered samples. Indeed, the density of the sintered composite was 97.3% of the theoretical density (lower than that of stainless steel alone), and the hardness was 4.6 GPa (higher than that of stainless steel).

#### 3.4.4. Composite Sintered from Granules Spheroidized with Preliminary Parameters

**Figure 13:**
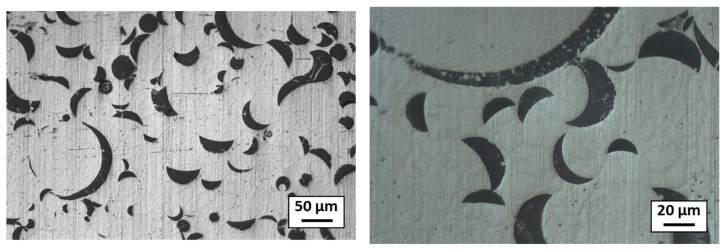
The optical microscopic microstructure of the composite sintered from the granules spheroidized with the preliminary parameters (parameter 3); alumina is in black and stainless steel is in grey.

Concerning the composite sample sintered from the spheroidized granules with preliminary parameters (Table 2, test 3), the microstructure is shown in Figure 13. Note the presence of large, moon-shaped alumina clusters (in black), which indicates that the alumina melted during the spheroidization process and clustered around the edges of the granules. The density of this composite was 98.1% and the hardness was 4.1 GPa. Compared with the simply atomized granules, there was a drop in hardness due to the poor homogeneity of the alumina. Spheroidization also reduced the presence of pores in the sintered pellet, explaining the increase in density.

#### 3.4.5. Composites Sintered from Granules Spheroidized with Optimal Parameters

**Figure 14:**
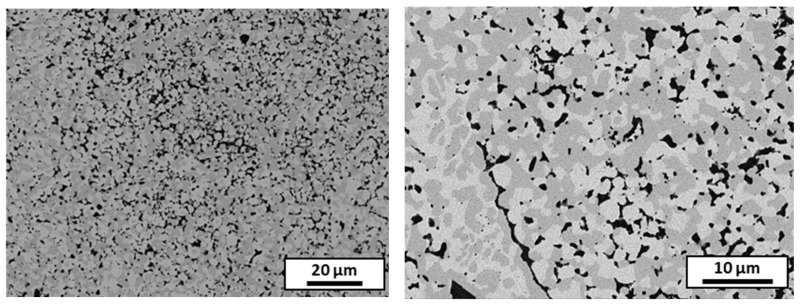
The SEM microstructure of the composite sintered from the granules spheroidized with the optimal parameters; alumina is in black and stainless steel is in grey.

The SEM images (Figure 14) reveal the homogeneous distribution of alumina (in black), as well as the austenitic and ferritic phases of the steel. The measured density of the sintered composite was 98.9% of the theoretical density, and the hardness was 5.4 GPa, which was higher than that of the stainless steel sample.

#### 3.4.6. Summary of Results for Sintered Samples

Table 5 summarizes the results of density and hardness obtained after sintering 80 vol% 316L/20 vol% Al_2_O_3_ composite samples from different raw powders: ball-milled mixture, spray-granulated powder, spray-granulated granules spheroidized with the preliminary parameters and spray-granulated granules spheroidized with the optimal parameters. These results are also compared with those for unreinforced sintered 316L powder. All these results were obtained with identical sintering parameters, as described in Section 2.5). The hardness of the composites depended on their porosity (it decreases when the porosity fraction increases) and on their microstructure (especially the distribution and the size of alumina particles). Therefore, in order to obtain a high hardness, the composite had to exhibit a high density and a homogeneous distribution of alumina particles. These conditions were fulfilled for the composite material sintered from a powder manufactured by a combination of spray granulation and spheroidization with optimized parameters.

To the best of our knowledge, no prior study has used the combined spray granulation and plasma spheroidization route for the fabrication of 316L/Al_2_O_3_ composites. The increase in hardness observed in our work exceeds that reported for 316L/Al_2_O_3_ composites processed by other techniques, including injection moulding [12], casting [10], hot pressing [6], laser powder bed fusion [11] and conventional sintering [5,16].

## 4. Conclusions

We have demonstrated the feasibility of a hybrid process that combines spray granulation, RF plasma treatment and SPS consolidation for the fabrication of 316L/Al_2_O_3_ composites. The optimization of the suspension formulation through precise adjustment of the pH, solid loading and selection of an appropriate dispersant resulted in homogeneous dispersion of the constituents, thereby limiting the agglomeration of alumina particles and enabling control over the granulometric distribution.

The optimization of the spheroidization parameters was essential for minimizing phase separation and encouraging partial recrystallization of the alumina onto the 316L. This was achieved by adjusting the plasma power, gas flow rates and powder injection position. However, the observed size-dependent behaviour highlights the complexity of heat transfer from the plasma to the particles. This requires adjustments to the granulometric distribution and more precise control of the processing parameters to prevent the over-melting of fine particles.

The composite samples, produced by SPS at a constant pressure of 64 MPa and under a controlled thermal cycle, exhibited a final density of 98.9% and a hardness of 5.4 GPa. Further microstructural analyses with SEM and microtomography revealed a uniform distribution of alumina within the 316L matrix, accompanied by a balanced distribution of austenitic and ferritic regions. This achieved an optimal balance between ductility and mechanical strength.

These results indicate that the developed approach opens up a promising pathway for the fabrication of metal matrix composites (MMCs) reinforced by ceramic particles. The combination of spray granulation and RF plasma treatment provides enhanced control over the metal/ceramic interfaces, while the characterisations of the SPS-prepared samples reveal a homogeneous phase distribution with excellent mechanical properties for applications requiring superior wear resistance and thermal stability.

## Figures and Tables

**Table 1:** Spheroidization parameters for preliminary tests.

	Preliminary Parameters						
	1	2	3	4	5	6	7
Power (kW)	40				30	30	20
Central gas (slpm)	Ar: 15						
Sheath gas (slpm)	Ar: 55/H_2_: 10					Ar: 55	
Carrier gas (slpm)	Ar: 5						
Injection probe position	Centre			−10 mm	Centre		
Powder feed rate (kg/h)	8	4	2				

**Table 2:** Spheroidization parameters for optimal treatment.

	Optimal Parameters
Power (kW)	20
Central gas (slpm)	Ar: 15
Sheath gas (slpm)	Ar: 55
Carrier gas (slpm)	Ar: 5
Injection probe position	−10 mm
Powder feed rate (kg/h)	4

**Table 3:** Slurry parameters.

**Slurry Batch**	316L (vol%)	Al_2_O_3_ (vol%)	Dispersant Ratio (wt%)	Solid Loading Ratio (wt%)	pH
1	80	20	2 PEG	40	7
2	80	20	2 PVA	30	11
3	80	20	0.5 PAA	50	11

**Table 4:** Density and size distribution characteristic values for the different granule batches.

1	2	3
D10: 1 µm	D10: 2 µm	D10: 2 µm
D50: 34 µm	D50: 26 µm	D50: 8 µm
D90: 266 µm	D90: 70 µm	D90: 36 µm
Density: 6.6 g/cm^3^	Density: 6.9 g/cm^3^	Density: 7.1 g/cm^3^

**Table 5:** Results of relative density and hardness of sintered materials.

		Relative Density (%)	Hardness (GPa)
Unreinforced 316L		99.5	3.7
Composite 80 vol% 316L/20 vol% Al_2_O_3_	Ball-milled mixture	91.8	4.3
	Spray-granulated granules	97.3	4.6
	Spheroidized granules with preliminary parameters	98.1	4.1
	Spheroidized granules with optimal parameters	98.9	5.4

## Data Availability

The original contributions presented in this study are included in the article. Further inquiries can be directed to the corresponding author.
